# Performance of two SARS-CoV-2 rapid antigen detection tests in resource limited settings, the case of Mali

**DOI:** 10.4314/ahs.v23i4.15

**Published:** 2023-12

**Authors:** Diallo Fatimata, Diarra Bassirou, Sarro Yeya dit Sadio, Tolofoudie Mohamed, Toure Mahamoudou, Diallo Dramane, Togo Antieme Congo Georges, Bane Sidy, Abdou Mohamed, Konate Mama Sy, Dicko Abdoul Razakou, Guindo Ibrehima, Saliba Katy Shaw, Kone Amadou, Mamadou Diakite, Doumbia Seydou

**Affiliations:** 1 University of Sciences Techniques and Technologies of Bamako, University Clinical Research Center (UCRC); 2 University of Sciences Techniques and Technologies of Bamako, Laboratory of Virology (LV); 3 National Institute of Public Health (INSP), Hippodrome, Bamako, Mali, Laboratory and Biomedical Research; 4 Ministry of Health and Social Development, Bamako, Mali, Health Referral Center Six; 5 Ministry of Health and Social Development, Bamako, Mali, Health Referral Center Four; 6 Division of Clinical Research, NIAID/NIH, Bethesda, MD, USA, Collaborative Clinical Research Branch

**Keywords:** SARS-CoV-2, rapid antigen tests, performance

## Abstract

**Introduction:**

While real-time reverse transcription PCR (RT-PCR) is the recommended laboratory method to diagnose severe acute respiratory syndrome Coronavirus 2 (SARS-CoV-2) infection, its use in resource limited settings can be difficult to maintain due to high testing demand and shortage of reagents. The aim of this study was to evaluate the performances of Realy Tech™ and Standard Q™ in comparison to RT-PCR in a relatively low COVID-19 prevalence setting, Mali.

**Methods:**

We conducted a cross-sectional study between January and April 2021 in Bamako and Kati regions to evaluate both rapid tests during a large SARS-CoV-2 prevalence study in Mali.

**Results:**

Of the 390 samples tested, the sensitivity and specificity of Realy Tech™ and Standard Q™ were 57.1% (95%CI: 44.1–69.2), 95.8% (95%CI: 93.1–97.5); 61.9% (95%CI: 46.8–75.0), and 94.1% (95%CI: 89.5–96.8) respectively. Using RT-PCR, the global prevalence of SARS-CoV-2 was 14.4% (56/390). In both rapid antigen tests, the performance was better when used in suspected patients compared to positive patients under treatment. Moreover, higher viral loads equivalent to Ct < 25 were associated with better detection rates.

**Conclusion:**

While waiting for more complete data, these preliminary studies suggest that Realy Tech™ and Standard Q™ should not be used alone for COVID-19 diagnosis in Mali.

## Introduction

Infection with severe acute respiratory syndrome Coronavirus 2 (SARS-CoV-2) results in COVID-19 disease, which can cause a spectrum of illness from asymptomatic to severe disease and death 1. The rapid detection and confirmation of a COVID-19 case is important not only for treatment of the patient but also for limiting human-to-human transmission through immediate case isolation and contact tracing. Increased risk of severity has been associated with older age, male gender, and chronic underlying health conditions 1-3. However, geographic and population variability in case rates and severity have been observed 4 5. Of particular note, there has been unexpectedly low disease mortality in Africa 6-8. WHO African region have register 8.5 million cases with around 171,331 deaths 9. While reported case and death numbers are lower than expected, and serosurveys from Kenya and Malawi suggest seroprevalence rates similar to those in areas experiencing high caseloads 6, 10.

While this may be partially due to insufficient molecular testing capacity, death related to COVID-19 in Africa have remained lower than expected and total excess mortality has not increased dramatically 11. It is unclear what the driving factors are for the variance in severity across populations. To increase the testing capacity mainly in Africa region, point-of-care rapid diagnostic tests (RDT) have been deployed.

Prior to the availability of rapid antigen tests, several African countries with different levels of COVID-19 burden reported limited performances of antibody tests 12, 13.

The SARS-CoV-2 Rapid Antigen Test is a reliable, rapid chromatographic immunoassay used in COVID-19 suspected cases for the qualitative detection of specific antigens of SARS-CoV-2 present in the human nasopharynx^14^. It identifies current infection during the acute phase of COVID-19, while the virus is still present in large quantities in the respiratory tract.

Mali, a developing country in West Africa, identified its first two cases in travellers from

France on 25 March 2020 is considered a relatively low prevalence setting of COVID-19. As of May 20th, 2022, the total numbers of COVID-19 cases were 31,048 resulting in 734 deaths and at least 301,454 reported contacts evaluated 15, 16. The use of this rapid test could be very useful because besides in the capital city, laboratory resources and capacity is limited. In addition, these rapid tests can also be an important tool for border (country entrances, land and air) control and surveillance. Thus, if implemented, these rapid tests will be a good alternative in sending samples from different areas to Bamako for testing, which leads to a long turnaround time. Before implementing, they need to be evaluated in each setting to determine the diagnostics performance. Thus, the goal of our study was to determinate the performance of Standard Q™ and Realy Tech™ rapid antigen tests using swabs from suspected and confirmed SARS-CoV-2 cases in Mali. Given that Malians and African individuals with mild infections might not seek medical care, and asymptomatic individuals are not usually screened, the prevalence of SARSCoV-2 including both RT-PCR and RDT could be more reliable estimate.

## Methods

### Study Sites and population

We conducted a cross-sectional study between January and April 2021 in three referral health centers in Bamako and two rural areas of Kati region. While Bamako, the capital city is the epicenter of the pandemic in Mali, Kangaba and Siby in Kati region are country-sides cities with very few caseloads of SARS-CoV-2 14. This evaluation of rapid antigen detection tests was part of a large SARS-CoV-2 prevalence study in Mali 17.

Study participants were either COVID-19 suspected cases or isolated confirmed positive patients under treatment and were informed and consented to be enrolled. The reason of inclusion of patients under treatment was to demonstrate that confirmed positive patients can be followed and treatment monitored by these rapid antigen tests. Although not active antibodies or antivirals, but with regards to Malian National regulation at that period, all positive SARS-CoV-2 patients were given according to weight and age and at free of charge the following dosage: Chloroquine (250mg) for 10 days; Azithromycin (500 mg) for 5 days and Vitamin C (1000 mg) for 10 days.

A paper based validated questionnaire was used to collect clinical and laboratory information from each participant and then double entered in an Excel spreadsheet to avoid error during data entry.

### Clinical specimens

Nasopharyngeal (NP) swabs were collected by trained medical personnel at referral health centers or treatment centers using sterile synthetic fiber swabs with plastic or wire shafts pre-wetted in Viral Transport Medium (VTM). If both NP and Oropharyngeal (Throat) (OP) swabs were collected, they were combined in a single tube to maximize test sensitivity. Samples were collected from consented household contacts of newly diagnosed SARS-CoV-2 positive patients irrespective of their clinical stage (symptomatic or not).

After carrying out rapid tests in the different study sites, all the samples for the RT-PCR were refrigerated and shipped to the University Clinical Research Center (UCRC) laboratory at the Faculty of Medicine and Dentistry, Point-G, Bamako, Mali within 6 hours of collection.

### Laboratory tests

Detection of SARS CoV-2 using the Standard Q™ and Realy Tech™ rapid antigen tests

Both “Antigen Testing” tests; Realy Tech™ COVID-19 Ag (Hangzhou Realy Tech Co. LTD, Hangzhou, China), and STANDARD Q™ COVID-19 Ag (SB Biosensor, SD Biosensor, Inc. C-4th&5th, 16, Gyeonggi-do, 16690 REPUBLIC OF KOREA) are rapid chromatographic immunoassays for the qualitative detection of specific antigens to SARS-CoV-2 nucleocapsid (N) present in human nasopharynx.

**Figure 1:**
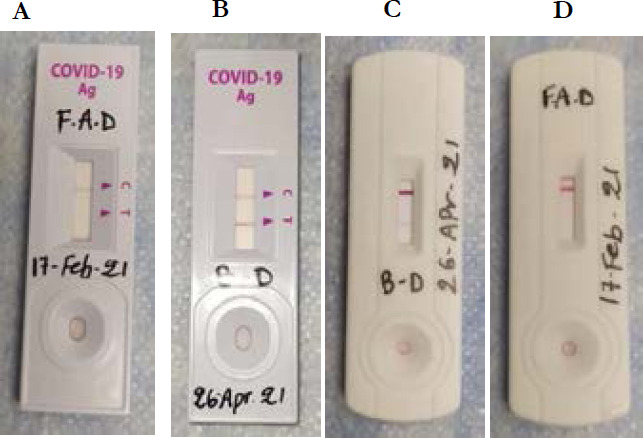
Interpretation of rapid SARS-CoV-2 antigen detection assay (Standard Q™ COVID-19 Ag Test: A & B), and (Realy Tech™ Ltd. C & D). Demonstration of A, a test which was interpreted as Standard Q™ negative SARS-CoV-2 antigen, B a test which was interpreted as Standard Q™ positive SARS-CoV-2 antigen, C a test which was interpreted as weak positive Realy Tech™ SARS-CoV-2 antigen, and D a test which was interpreted as Realy Tech negative SARS-CoV-2 antigen

These tests were performed by healthcare workers or laboratory personnel only, as an aid to early diagnosis of SARS-CoV-2 infection in patient with and without clinical symptoms with SARS-CoV-2 infection 18 (Figure 1). Both tests were run according to manufacturer's recommendations. Briefly, after a NP sample collected, the swab used was put into directly the buffer, mixed and thereafter drops were deposit into the respective cassettes. Tests were read between 15-30 minutes and results interpreted as: positive (appearance of 2 bands control and test bands), negative (appearance of a control band only) (Figure 1), and invalid and/or uninterpretable results (appearance of test band only without control, and/or absence of both bands).

Following the rapid test, the remaining samples were stored at 4°C before transportation to the University Clinical Research Center (UCRC) for RT-PCR on the same days. All RT-PCR-positive cases were referred to the COVID-19 treatment center and their contacts were identified and followed up based on the national COVID-19 management guidelines.

### Detection of the SARS CoV-2 virus by RT-PCR

Sample inactivation was performed under a class II biosafety cabinet in Biosafety level 3 plus (BSL-3+) laboratory at UCRC. An appropriate amount of the clinical specimen (range 100-140 µl) was added to a tube containing the inactivation mixture for the required contact time (between 10-20 minutes) followed by vortex and incubation. Thereafter, the tubes were spray-disinfected with alcohol from the outside and removed from the cabinet and transferred to the BSL-2 Molecular Biology Laboratory. RNA then extracted from the inactivated samples using Qiagen QIAamp® Viral RNA mini kit. RT-PCR was conducted using ARGENE® SARS-CoV-2-R-GENE® (BioMérieux, Lyon, France) kits according to manufacturer's protocol 4, and results were reported as: negative, positive or indeterminate based on the presence of N and/or E Genes of SARS-CoV-2 according to manufacturer's guidelines. Given the no replicative signals of intermediate replicative RNAs associated with non-in-fectiousness of individuals when SARS-CoV2 RT-PCR cycle threshold value (Ct) is above 33 in previous studies, we considered positive, only samples with RT-PCR Ct value cut-off less or equal to 33 19, 20. Moreover, as per Institutional guidelines, all positive and indeterminate results were repeated once.

### Samples size estimation

All the screening tests performance (sensitivity, specificity, predictive positive and predictive negative values) were used to estimate the performance of both Realy Tech™ and Standard Q™ Ag tests. RT-PCR was considered as the gold standard for this evaluation, therefore positive and negative samples by RT-PCR were considered to be true positive and true negative samples, respectively.

With a predicted performance of 95% of sensitivity, 85% of specificity, a power of 80% and 5% error margin, the estimated sample size was estimated to 299 participants.

### Statistical analyses

Descriptive statistics were used to describe patient socio demographic information. Data were presented in number and percentage. Sensitivity, specificity, positive predictive value (PPV), negative predictive value (NPV) was calculated using OpenEpi tool in Epi Info version 7.2. Interquartile range (IQR) was calculated for 25% and 75%.

### Ethics statement

The clinical specimens included in this manuscript were collected under public health surveillance and not as human subject research. Thus, submission to institutional review boards was not applicable. Participants were explained, and they consented before.

## Results

Three-hundred and ninety nasopharyngeal swabs were collected from 390 patients.

**Figure 2:**
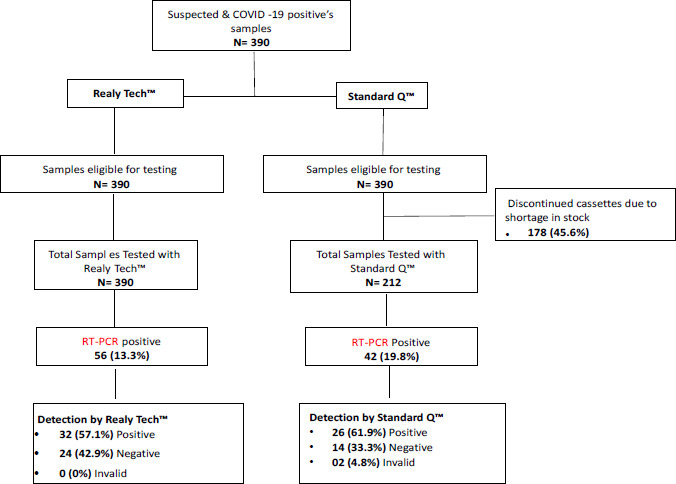
Flow Chart of study of suspected COVID-19 samples included in the final analysis in evaluating Realy Tech and Standard Q, rapid antigen tests in Bamako and Kati Region, Mali, in 2021

Of the 390 samples, 324 (83%) were from suspected patients and 66 (17%) from confirmed SARSCoV-2 positive patients under treatment. The median age of patients was 36 years (IQR, 28-46 years), and the sex ratio was 2.1 (265 men and 125 women). Two-hundred and ninety-eight (76.4%) of samples were collected in Bamako region. Three-hundred and sixty-seven (94.1%) of total patients were asymptomatic. Using RT-PCR, the prevalence of SARS-CoV-2 was 14.3% (56/390), but 11.8% (46/390), and 16.9% (36/212) for rapid tests Realy Tech™ and Standard Q™ respectively (Figure 2). The median Ct values of SARS-CoV-2-positive patients was 28 (IQR, 22-30 cycles).

### Diagnostic performances of COVID-19 Ag Rapid tests

Among the 56 samples positive by RT-PCR, Realy Tech™ detected 32 (Table 2), and its overall performance was 57.1% (95% Confidence Interval (CI): 44.1-69.2), 95.8% (95% CI: 93.1-97.5), 69.6% (95% CI: 55.2-80.9), 93.0% (95% CI: 89.8-95.3), and 90.3% (95% CI: 86.9-92.8) for sensitivity, specificity; positive predictive value (PPV); negative predictive value (NPV); and diagnostic accuracy respectively.

In addition, when samples were broken out by RT-PCR Ct value, the sensitivity of Realy Tech™ Ag test was 88.2% (95% CI: 65.7-96.7) (Ct <25, n= 15), and 65.8% (95% CI: 49.9-78.8) (Ct < 30, n = 25) (Table 1 & Table 3). Standard Q™ detected 26 of the 42 positive samples (Table 3), and the overall performance was 61.9% (95% CI: 46.8-75.0) and 94.1% (95% CI: 89.5-96.8) ,72.2% (95% CI: 56.0-84.2), 90.9% (95% CI: 85.7-94.3) and 87.7% (95% CI: 82.6-91.5) for sensitivity, specificity; PPV; NPV; and diagnostic accuracy respectively (Table 2 & Table 3). Moreover, when analysed by Ct value on the RT-PCR, the sensitivity of the Standard Q™ was 100% (95% CI: 72.3-100) (Ct < 25, n=10), and 88.9% (95% CI: 67.2-97.9) (Ct < 30, n=18).

**Table 1:** Results of Realy Tech COVID-19 antigen test

		RT-PCR	
**Realy Tech™** COVID-19 Ag test		Detected	Not Detected
	Detected	32	14
	Not Detected	24	320

RT-PCR= Real-time reverse transcription polymerase chain reaction

**Table 2:** Results of STANDARD Q COVID-19 antigen test

		RT-PCR	
**Standard Q™** COVID-19 Ag test		Detected	Not Detected
	Detected	26	10
	Not Detected	16	160

RT-PCR= Real-time reverse transcription polymerase chain reaction

**Table 3:** Diagnosis Performances of COVID-19 Ag Rapid tests

		Diagnosis Performances			
		
		Sensitivity (%) 95% [CI]	Specificity (%) 95% [CI]	PPV (%) 95% [CI]	NPV (%) 95% [CI]
Overall	Realy-Tech™ (N= 390)	57.1	95.8	69.6	93.0
		[44.1-69.2]	[93.1-97.5]	[55.2-80.9]	[89.8-95.3]
	Standard Q™ (N=212)	61.9	94.1	72.2	90.9
		[46.8-75.0]	[89.5-96.8]	[56.0-84.2]	[85.7-94.3]
	
Suspected cases	Realy-Tech™ (N= 324)	68.6	97.6	77.4	96.3
		[52.0-81.6]	[95.1-98.8]	[60.2-88.6]	[93.4-97.9]
	Standard Q™ (N=175)	65.4	97.3	80.1	94.2
		[46.2-80.6]	[93.3-98.1]	[60.0-92.3]	[89.3-96.9]
	
Under Treatment	Realy-Tech™ (N= 66)	38.1	84.4	53.3	74.5
		[20.8-59.1]	[71.2-92.3]	[30.1-75.2]	[61.1-84.5]
	Standard Q™ (N=37)	56.3	71.4	60.0	68.2
		[33.2-76.9]	[50.0-86.2]	[35.8-80.2]	[47.3-83.6]
	
With Symptoms*	Realy-Tech™ (N= 23)	100	100	100	100
		[64.6-100]	[80.6-100]	[64.6-100]	[80.6-100]
	Standard Q™ (N=13)	100	100	100	100
		[43.8-100]	[72.3-100]	[43.8-100]	[72.3-100]

Oropharyngeal	Realy-Tech™ (N= 63)	58.8	82.8	80.0	63.2
		[42.2-73.6]	[65.5-92.4]	[60.9-91.1]	[47.3-76.6]
	Standard Q™ (N=61)	62.5	69.0	69.0	63.0
		[45.3-77.1]	[50.8-83.0]	[50.8-83.0]	[45.3-77.1]
Oropharyngeal + Nasopharyngeal	Realy-Tech™ (N= 327)	54.6	97.1	57.1	96.7
		[34.7-73.1]	[94.5-98.4]	[36.6-75.5]	[94.1-98.2]
	Standard Q™ (N=151)	60	99.3	85.7	97.2
		[31.1-83.2]	[96.1-99.9]	[48.7-97.4]	[93.1-98.9]

***CI:*** *confidence Interval;* ***PPV:*** Positive Predictive Value; **NPV:** *Negative Predictive Value*

* ***Symptoms*** *include mainly* cold, cough, *but also rhinitis, back and muscle pain*, headache, fatigue, sneezing, anosmia, and rhinitis.

### Performances of COVID-19 Ag Rapid tests by patient's category

Realy Tech™ and Standard Q™ were evaluated in suspected patients and confirmed SARS-CoV-2 positive patients under treatment for screening new patients and following positive patients for clearance purposes. We found that both tests performance was higher in suspected patients compared to the overall performance. Moreover, the test-performance of both tests decrease drastically in patients under treatment (Table 3).

In addition, while categorizing by symptoms, the performance of both RDT was significantly improved up to 100% concordance with real-time RT-PCR if symptoms appeared (Table 3). But the performance was not improved when patients were categorized by collection swab type (Table 3).

## Discussions

Laboratory capacity and accurate point-of-care technologies are critical component public health response to COVID-19 pandemic through timely diagnosis of viral infection, patient monitoring, as well as epidemiologic surveillance of SARS-COV-2. It provides objective biological data to clinicians and public health leaders for appropriate decision-making. The use of rapid test will be more important in a setting with limited resources where sophisticated laboratories are often present only in large cities. In Mali for instance, some cities must send samples more than 1,500 km to be tested, leading to a long turnaround time, delay in patient management, and high risk of household contamination.

The rapid confirmation of a COVID-19 case is important not only for treatment of the patient but also for limiting human-to-human transmission through immediate case isolation and contact tracing. Thus, rapid antigen tests have been developed to screen early before RT-PCR results. In a resource constrained settings like Mali, where access to molecular testing reagents and supplies has been difficult during this pandemic outbreak, rapid, easy to use antigen tests would provide an advantage for pandemic response.

We report here the performance of two rapid antigen tests, Realy Tech™ and Standard Q™, compared to RT-PCR, leveraging on our emerging infectious pathogens diagnosis capacity built from 2015's Ebola outbreak, collaboration and research partnership that have been instrumental in developing molecular detection of SARS-CoV-2 virus in Mali 21-23.

Overall, we had a limited performance of both rapid antigen tests for this study in Mali. Although previously poor sensitivity has been reported for Standard Q™ at 17.5% (95% CI, 8.8–32.0%) in South Korea 24, but a relatively low sensitivity at 58.1% (95% CI 42.1–73.0), and 70.0% (95% CI: 60–79) was seen in Serbia and Uganda respectively for the same SARS-CoV-2 antigen test 25, 26. Moreover, we had also similar poor sensitivity at 30.2% (95% CI: 21.7-39.9 using another rapid test, the COVID-19 Respi-strip Antigen test in Belgium 27. In contrast, Chaimayo et al, using Standard Q™ found a greater sensitivity and specificity of 98.33% (95% CI: 91.06-99.96); 98.73% (95% CI: 97.06-99.59) respectively 28. This performance evaluation occurred in Thailand which had higher prevalence compared to Mali and may explain the difference. It will be important to continue the surveillance of the test performance during an outbreak of this high transmissible virus in our setting and as new variants arise. Moreover, seven distinct variants were identified in our laboratory during that period including three variants of concerns (VOC): Delta, Beta, and Alpha; two variants of interest (VOI): Eta and 20B; and two variants under monitoring (VUM), 19B, and 20A (Manuscript under review).

On the other hand, we had a ‘relatively moderate’ performance when using only in suspected cases compared to patients under ‘treatment’. This is due probably to the fact that, patients under ‘treatment’ may have low viral load (increased Ct value) compared to new patients. In addition, the poor performance of both tests in patients under ‘treatment’ may definitively exclude their use in determining clearance in patients under ‘treatment’. It could also be the opposite: these non-specific treatments had no impact on viral loads, and thus tests performed poorly. In addition, although only 6% of study participants were symptomatic, but both tests had 100% concordance with TR-PCR when symptoms were present. But at the same time, the performance was not improved when patients were categorized by collection swab type.

In spite of the ease and fast achievement of the RDT compared with molecular techniques, the analytical performances of these rapid antigenic tests depend on different factors such as viral load, the stage of infection, the quality of the specimen, test performer, and the prevalence of COVID-19 in the setting of the evaluation. Thus, in Mali with a relatively low prevalence setting of COVID-19, we found a limited sensitivity of both anti-genic tests. Sequencing data already showed the multiple and independent introductions of SARS-CoV-2 in Mali which could partially explained this situation 29.

Moreover, when viral load was higher (as proxy by the Ct value on the RT-PCR), Ct < 25, the sensitivity improved; 88.2% for Realy Tech™ and 100% for Standard Q™. This is potentially useful as samples with high viral load are more likely to produce isolatable virus, indicating patients would potentially be infectious. If these patients were able to be diagnosed right after the sample collection and start treatment and isolation, it would prevent more transmission of the virus to their contacts.

On the other hand, sensitivity declines when the viral load decreases equivalent to Ct values over 30, which is often the case in patients suffering of COVID-19 in Mali given the overall median Ct value of 28, and the high prevalence of asymptomatic patients. In addition, the limited sensitivity of both rapid antigen tests could lead to false negative results, which in this pandemic will not help identifying cases, mainly if there is suspicion of low number of samples for testing as an explication of our relatively low prevalence rate.

Though, the sensitivity of both tests was limited, they could still be used in resource limited settings such as Mali. In fact, the Mali National COVID-19 guidelines suggest to screen first with RDT, and test RDT negative patients with RT-PCR for confirmation. Thus, positive-RDT patients can directly begin treatment and isolation to limit the spread of disease. These RDTs are suitable for airport and road suspected travellers as tool for decision making in area where RT-PCR turnaround time may be very long. Moreover, we didn't have an issue with the feasibility and the implementation of these RDTs, thus these characteristics in addition to sensitivity and specificity data should be considered while analysing the test performance during this long pandemic 30.

This is one of the first studies in our setting evaluating the use of RDT in SARS-CoV-2 diagnosis and should lead to important recommendations in implementing these rapid tests in the peripheral areas where RT-PCR is not available, and if patients had symptoms. The strength of this study is the real-world situation evaluation. The study population was tested directly in the sample collection site.

## Limitations

Though, our study had some limitations. First the study population does not fully represent the general population of Mali, as there is no general population screening for COVID-19, and we may have not reached some COVID-19 patients. Second, the patients were relatively young, but this is reflecting the Malian general population, and we may not know how better the RDT performance is in older vs. young age in this population.

## Conclusion

Despite these limitations, this prospective study provides an ‘unbiased’ structure of COVID-19 population of Mali including volunteers from both epicentre and less transmission areas. While waiting for more complete data, this preliminary evaluation study suggests that Realy Tech™, and Standard Q™ COVID-19 Antigen test should not be used alone for COVID-19 diagnosis in Mali for but should always be followed by a RT-PCR or other diagnosis means for better decision. At the same time, they had better performance on symptomatic patients.

## Data Availability

Data will be made available upon request.
